# Evaluation of stream visual assessment protocol with measured water quality parameters in urban streams

**DOI:** 10.1371/journal.pone.0351972

**Published:** 2026-06-25

**Authors:** Caleb McMurray, Abigail C. Finch, Kennedy Jones, Eli Keaton, Hyde Parkinson, Norah Patterson, Jennifer James, JoAnn Burkholder, Ana Meza-Salazar, Erin A. McKenney

**Affiliations:** 1 Department of Biological Sciences, NC State University, Raleigh, North Carolina, United States of America; 2 Department of Applied Ecology, NC State University, Raleigh, North Carolina, United States of America; 3 Center for Applied Aquatic Ecology, North Carolina State University, Raleigh, North Carolina, United States of America; University of Bucharest: Universitatea din Bucuresti, ROMANIA

## Abstract

Accurate assessment of stream health is important due to the numerous known influences of habitat, including water quality, on aquatic ecosystems as well as human health. The Stream Visual Assessment Protocol (SVAP) of the Natural Resources Conservation Service is an accessible resource for resource managers and the citizenry that provides a rapid “snapshot” assessment of general stream health, based on visual physical characteristics. This study evaluated whether SVAP scores can be used to infer water quality conditions. We assigned SVAP scores for four urban streams in the southeastern U.S. Piedmont region using ten metrics, and assessed whether the scores were strongly correlated with measured data for four physical features (depth, flow rate, canopy cover, water temperature) and seven water quality parameters (pH, dissolved oxygen concentration and percent saturation, conductivity, total dissolved solids, turbidity, and *Enterococcus*). The SVAP scores were strongly correlated with measured physical characteristics, but there were few significant correlations between SVAP scores and water quality data. The findings suggest that the SVAP should not be used to infer water quality conditions, which are critically important to stream habitat health.

## Introduction

Water quality has important implications for aquatic life and human use [1]. Poor water quality may prevent potable use of a freshwater source, limit recreation, and decrease aquatic biodiversity [2,3]. Water quality in freshwaters has been historically assessed using various metrics such as pH, conductivity, turbidity, total dissolved solids, dissolved oxygen, nutrient concentrations, algal biomass as the indicator pigment chlorophyll *a*, and pathogenic bacteria or their indicators [3,4]. Features such as terrestrial canopy cover [5], temperature, flow rate [3], and water depth [6] can provide insights about physical characteristics that affect stream biodiversity.

The United States Department of Agriculture (USDA) Stream Visual Assessment Protocol (SVAP) was developed as an initial screening technique for the general condition of wadeable streams based on visible physical features, without actual water quality measurements [7,8]. The SVAP protocol includes written instructions to assign scores from 10 (excellent) to 1 (poor) for up to 15 elements, using a rubric of narrative descriptions. As such, the SVAP does not require expensive materials or specialized training, and it has been implemented widely across the U.S. [2,9,10] and in other nations (e.g., [11–13]).

The SVAP has mostly been compared to macroinvertebrate metrics indicative of stream health [2,10,13]. Previous field researchers have reported that SVAP scores were weakly and inconsistently correlated with macroinvertebrate indices in various geographic locations [2], and suggestions have been made for strengthening the utility of the SVAP to more closely parallel inferences from metrics for general macroinvertebrate community health [10,14]. In the SVAP, general water quality is inferred by appearance (color and turbidity) and nutrient enrichment is inferred from obvious algal growth. However, the SVAP has not been compared to actual water quality data for parameters such as dissolved oxygen, conductivity, or total dissolved solids, so its utility for indicating general water quality conditions is unknown. Correlation to water quality is important to complement conventional macroinvertebrate bioindicators, especially in urban streams where conditions are generally poor for sensitive macroinvertebrate taxa [15,16].

Given the altered state characteristic of urban streams [17] and often-rapid urbanization rates [18], it is increasingly important to identify accurate and accessible alternative protocols to monitor the biological and chemical health of urban streams [19]. We therefore assessed the potential of the Natural Resources Conservation Service (NRCS) SVAP for rapid assessment of general water quality of four urban streams by comparing the scores to measurements for several physical characteristics and water quality parameters. We expected that visual stream characteristics would correlate well with the general conditions indicated by the water quality data.

## Methods

### Study area and streams

The four streams were in the Piedmont region of North Carolina, USA, within the greater Raleigh urban area, which has sustained the second highest growth of any urban area in the United States in the last decade. The City of Raleigh grew from 467,911 people in 2020 to 499,825 people in 2025, a 6.8% increase [20]. Urbanization is rapidly transforming watersheds across the Piedmont in central North Carolina, with implications for urban watershed hydrology and water quality (e.g., [21,22]). Compared to fully forested watersheds, urbanized Piedmont watersheds discharge 75% more stormflow per precipitation event and perform 19% lower evapotranspiration rates [23], slowing recovery of urban watersheds after storm events. A comparison of Bolin Creek to two reference streams in Chapel Hill, NC suggests that urban runoff from impervious surfaces drives water quality degradation as measured by conductivity, turbidity, and nutrient concentration [24].

**Fig 1:**
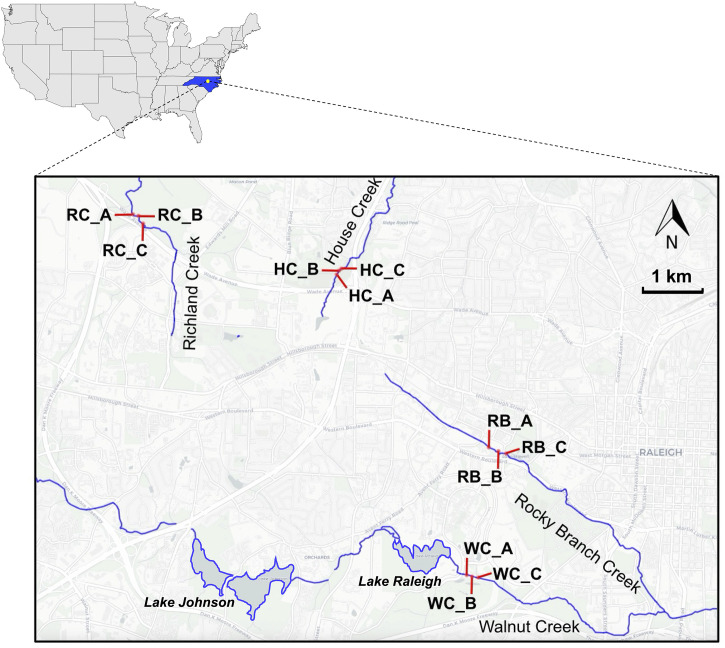
Map showing the study area and sample sites on Richland Creek, House Creek, Rocky Branch Creek, and Walnut Creek in the upper Neuse River watershed, Raleigh, NC. Lakes Johnson and Raleigh are run-of-river impoundments along Walnut Creek; also note that the apparent breaks in Walnut Creek are locations where the stream went under a roadway or culvert. Maps were created using the maps, osmdata, leaflet, and ggplot2 packages in **R.** See Table 1 for specific site locations.

We assessed the four urban streams during a four-week period (Table 1, Fig 1) from 24 October to 14 November 2024. We received permission from Rachel Woods (NC Museum of Art) to access House Creek and from Laxmi Parajuli (Schenck Forest) to access Richland Creek; Walnut Creek and Rocky Branch Creek were accessed on NC State University campus. Permits were not required to collect the data for this study because no specimens were removed and the stream assessment was not disruptive. Each stream was analyzed at three different 5-m-length sites, spaced at least 100 m apart. All locations at each stream were assessed twice on randomized dates in an attempt to avoid weather as a confounding variable. Data were collected from Rocky Branch (RB) and Walnut Creek (WC) on 24 October, Rocky Branch and House Creek (HC) on 31 October, Richland Creek (RC) and Walnut Creek on 7 November, and Richland Creek and House Creek on 14 November.

**Table 1:** Stream locations and brief description of areas sampled.

Sample site	Latitude	Longitude	Brief description
RC_A	35.8148168	−78.7334343	~15m from Wade Ave with bank reinforcement and a culvert present
RC_B	35.81462	−78.73253	~80m from Wade Ave with a large rocky beach present, widest site of stream measured
RC_C	35.8134789	−78.7316711	~35 m from Wade Ave with steep natural embankments on both sides and culvert upstream
HC_A	35.8069382	−78.6961624	Heavily wooded riparian zone with several fallen trees present, very shallow
HC_B	35.8066801	−78.6965828	Narrow embankments with rocky beaches on either side
HC_C	35.8061510	−78.6970203	Detritus leaves and rocks cover majority stream surface area, closest site to footpath
RB_A	35.7801484	−78.6667100	Runoff from Pullen Rd and Western Blvd with grass embankments and heavy foot traffic
RB_B	35.7804724	−78.6681380	Large riparian zone, furthest from footpath, plenty of room for flooding
RB_C	35.7810806	−78.6698261	Steep embankments, wedged between footpath and Dail Soccer Field
WC_A	35.7625558	−78.6737777	Pipe runs over site with large rocks impeding flow, closest to foot traffic and Main Campus Dr
WC_B	35.7623373	−78.6728399	~45 from Lonnie Pool golf course
WC_C	35.7621719	−78.6717613	Heavily wooded with steep embankments, ~ 260m from Main Campus Dr

House Creek originates from a small reservoir southwest of the intersection of Interstate 440 and Wade Avenue in western Raleigh, North Carolina. The stream flows north for ~1.9 km before joining another first-order tributary from a reservoir near the east border of UNC Rex Hospital. The second-order House Creek tributary then parallels Interstate 440 for approximately 3.0 km before discharging into Crabtree Creek. Despite its position within a heavily urbanized corridor and its repeated crossings beneath the interstate, House Creek maintains a relatively intact riparian zone. This vegetation contributes canopy cover and woody debris inputs, providing important ecological functions and supporting in-stream habitat.

Richland Creek arises from a pond located on the North Carolina State Fairgrounds south of Carter–Finley Stadium. The stream flows north through Schenck Forest and along the southern boundary of William B. Umstead State Park before joining Crabtree Creek. With the confluence of two unnamed tributaries along its course, Richland Creek is classified as a second-order tributary. In May 2023, sections of the streambank near the sampling sites were reinforced to mitigate stormwater-induced erosion caused by runoff from adjacent Wade Avenue [25]. While the stream originates in what is now a highly developed area, much of its length flows through protected forested land, offering ecological benefits comparable to those of House Creek.

Rocky Branch Creek originates near the southern end of Meredith College campus, which forms the upper extent of the watershed by elevation. Stormwater from the campus is conveyed via a system of ponds and open channels to a set of culverts beneath Hillsborough Street and the railroad, where it is joined by stormwater flow from a small area west of I-440 and transitions to a natural stream channel east of Beryl Road and flows southeast into North Carolina State University’s Central Campus at the intersection of Gorman Street and Sullivan Drive [26]. The stream continues through campus before exiting near the southeastern edge and following Western Boulevard until joining Walnut Creek northwest of the Interstate 40 and Hammond Drive intersection, where it functions as a first-order tributary. Along its course, Rocky Branch traverses extensive culverts and stormwater infrastructure, with an estimated ~97 km of stormwater pipes present within its watershed (~27 km of which are maintained by the city of Raleigh). In addition, the Rocky Branch Creek Greenway parallels much of the stream length through five Raleigh city parks. Together, these factors have contributed to a highly urbanized stream corridor characterized by limited riparian zones and reduced floodplain connectivity.

Walnut Creek, a fourth-order stream [27], originates from Maynard Pond in a residential neighborhood of Cary, North Carolina, and flows eastward through southern Raleigh before joining the Neuse River near the intersection of Poole Road and Barwell Road. Along its course, Walnut Creek traverses several impoundments including Lakes Cramer, Johnson, and Raleigh, and it receives inflow from multiple smaller tributaries. Because of its length and catchment size, Walnut Creek traverses a range of land-use settings including residential areas, public conservation lands, and the Centennial Campus of North Carolina State University. Consequently, its ecological condition reflects a composite of both beneficial and detrimental characteristics as observed in the other streams.

### SVAP application

**Fig 2:**
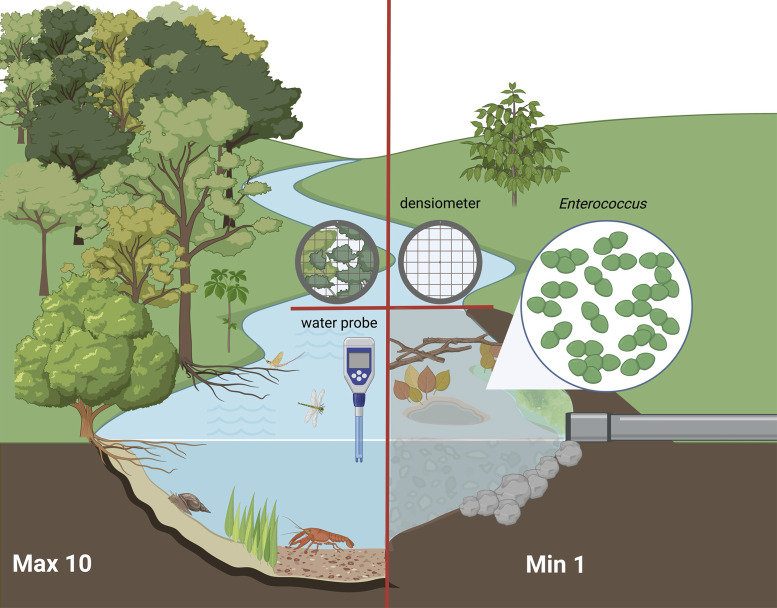
Representation of stream characteristics with maximum (10) and minimum (1) scores for each of ten factors on the stream visual assessment protocol (SVAP). The spherical densiometer, water probe, and *Enterococcus* represent physical characteristics and water quality parameters. Created in BioRender. Jones, **K.** (2026) https://BioRender.com/c4y8r1i.

Each stream was assessed following the SVAP [7], and was assigned a score ranging from 1 to 10 (Fig 2). Three researchers scored each stream site based on ten factors (channel condition, hydrologic condition, riparian zone, bank stability, water appearance, nutrient enrichment, barriers to fish movement, instream fish cover, pools, invertebrate habitat) using SVAP guidelines. The team used the SVAP worksheet to score each factor from 1–10 for evaluation, and then discussed their individual assessments to arrive at a consensus score for each factor at each site. A short description of the SVAP guidelines, including an explanation of our interpretation of each of the scores, is provided in Supplementary Table 1. We randomized which three researchers completed the SVAP at each site on each day to avoid scoring bias. We averaged the scores across all ten factors to calculate an overall health score for each stream site, per SVAP guidelines.

### Measured physical characteristics

Air temperature (°C) and relative humidity (%) were measured at each site using a Kestrel 3000 Weather Meter (Kestrel Instruments, Boothwyn, PA, USA). Water temperature (°C) was measured using a YSI ProSolo ODO Optical Dissolved Oxygen Meter (Xylem Inc., Washington DC, USA). Water depth (cm) was measured with a meter stick at three points (near left bank, mid-stream, and near right bank) and averaged for each site. Flow rate (m/s) was measured using a meter stick, a ping pong ball, and a stopwatch (*sensu* [28]). Canopy cover by terrestrial vegetation was measured with spherical crown densiometers (model 43887, Forestry Suppliers, Jackson, MS, USA).

### Measured water quality parameters

We also collected water quality data at each stream site. Dissolved oxygen (DO, mg/L and percent saturation, %) was measured using a YSI ProSolo ODO Optical Dissolved Oxygen Meter (Xylem Inc., Washington DC, USA). Conductivity (μS/cm), total dissolved solids (TDS, mg/L), and pH were measured with an Apera PC60 pocket water probe (Apera Instruments, Columbus, OH, USA). Turbidity (NTU) was measured with a Hatch 2100P portable turbidimeter (Hatch, Loveland, CO, USA).

We quantified *Enterococcus* bacterial densities in each stream using composite samples collected from each of the three sites and pooled. One vial of Enterolert reagent was mixed into each 100-mL sample; samples were sealed in a 49-well Quanti-tray/2000 (IDEXX Laboratories, Westbrook, Maine, USA), and trays were incubated at 41.0 °C for ~24 hr (Fisherbrand Isotemp Microbiological Incubator, Hampton, NH, USA). The Quanti-trays were then placed into a Spectroline model CM-10 fluorescence analysis cabinet (Spectronics Corporation, Westbury, NY, USA). Glowing cells indicating the presence of *Enterococcus* were marked, and an IDEXX Quanti-Tray*/2000 most probable number (MPN) table was used to estimate the MPN 100 mL^-1^.

### Hypothesized relationships between SVAP and water quality metrics

We considered ten SVAP metrics, four measured physical environmental features, and seven water quality parameters in this assessment. Prior to the analysis, we hypothesized positive or negative relationships suggested by significant correlations between SVAP metrics and measured physical environmental characteristics or water quality parameters (Table 2). Since many of the factors are known to interact [28–30], the predicted (expected) effects in various comparisons were both positive and negative depending on the parameter levels.

**Table 2:** Hypothesized relationships between SVAP factors and water quality metrics, and rationale for these predictions (concepts taken from [28–31]).

SVAP metric	Measured physical characteristic(s)	Water quality parameter(s)	Correlation	Rationale
Channel condition	Average depth, flow rate	-----	Negative	Shallow, slowly moving waters can promote noxious algal/ plant growth. Faster flow can cut a deeper channel and increase shading and bank erosion/instability.
	-----	Total dissolved solids (TDS), turbidity	Negative/ Positive	Can increase TDS and turbidity or, at higher levels, can dilute them.
Hydrologic alteration	Average depth, Flow rate	-----	Negative	Shallow waters are more prone to drying during droughts. More flooding occurs when the channel is not incised (shallow stream) or in slowly moving waters (storm events have greater impact).
Riparian zone	Canopy cover	-----	Positive	Riparian plant zones increase shading and allocthonous inputs (energy source). Intact riparian zones reduce runoff and increase pollutant filtration.
		TDS, turbidity, *Enterococcus*	Negative	
Bank stability	Canopy cover	-----	Positive	Terrestrial vegetation increases bank stability, reducing erosion and decreasing pollutant inputs.
		TDS, Turbidity, *Enterococcus*	Positive	
Water appearance (basis, visual appearance, e.g., color and transparency)	Flow rate	-----	Positive	Increased flow can clear turbidity and algal growth.
		Dissolved oxygen (DO),	Positive/ Negative	Increased flow can increase DO (though if too high, may appear cloudy due to supersaturation).
		TDS, Turbidity, Conductivity, *Enterococcus*	Negative	Particulates and bacterial growth cloud water.
Nutrient enrichment (basis: apparent algal and plant growth)	Canopy cover	-----	Positive	Bank vegetation filters nutrient inputs in runoff. Excess nutrients stimulate noxious algal and bacterial growth, causing harmful DO and pH “swings” and increased turbidity.
		DO, pH,	Positive/ Negative	
		Turbidity, *Enterococcus*	Positive	
Barriers to fish movement	Flow rate	-----	Positive/ Negative	Barriers can slow water flow and its abrasive effects, improving fish habitat. However, barriers can also increase temperature and decrease DO due to stagnation.
		Water temperature, DO		
Instream fish cover	Depth	-----	Positive	Gentle flows and increasing depth can maintain debris used as valuable habitat. Increasing flow tends to increase DO but can scour branches and other habitat.
	Flow rate		Positive/ Negative	
	Canopy cover	-----	Positive	Can contribute to instream fish habitat, cooler temperatures, and improved general water quality.
	-----	Water temperature	Negative	Increasing fish cover can decrease water temperature via shading.
	-----	DO	Positive	Healthy algal/plant cover can increase DO while also decreasing turbidity and TDS.
	-----	Turbidity, TDS	Negative	
Pools	Average depth	-----	Negative	Pools in moderately deeper areas of streams can have cooler temperatures that retain more DO.
		Water temperature	Negative	
		DO	Positive	
Invertebrate habitat	Flow rate	-----	Positive/ Negative	Slowly moving water maintain physical habitat that can be dislodged under faster flow.
	Canopy cover	-----	Positive/ Negative	Canopy cover can provide debris that becomes valuable habitat. However, dense cover can block light available for beneficial algal/plant growth.
	-----	Water temperature, DO, Conductivity, pH	Positive/ Negative	These parameters can positively or negatively influence habitat, depending on the level or concentration and optima for sensitive taxa.
	-----	Turbidity, TDS, *Enterococcus*	Positive	Minimal conditions for these parameters enhance invertebrate habitat.

### Statistical analysis

Because we measured *Enterococcus* from pooled samples for each stream, we first averaged the SVAP scores and water quality measurements across the three sites in each stream, to compare a single composite value for each measure. Spearman’s Rank Correlation Test was used to quantify the correlation and its strength between each of the averaged SVAP scores and each mean measurement for the water quality parameters for each visit (n = 2), across streams (n = 4). Spearman’s correlation converts raw data into ranks and then calculates the correlation coefficient to quantify the strength (0–1) and direction (positive or negative) of the relationship (Sokal and Rohlf 2012). Importantly, Spearman’s correlation is a non-parametric test, making it robust to outliers and data that are not normally distributed. We analyzed data and created graphs in R Studio (R version 4.3.3, R Project for Statistical Computing, Vienna, Austria) using the packages “tidyverse,” “ggcorrplot,” “corrplot,” and “Hmisc.” The data and Rscript are available in the Dryad data repository (https://doi.org/10.5061/dryad.7m0cfxq89).

## Results

### SVAP metrics

**Fig 3:**
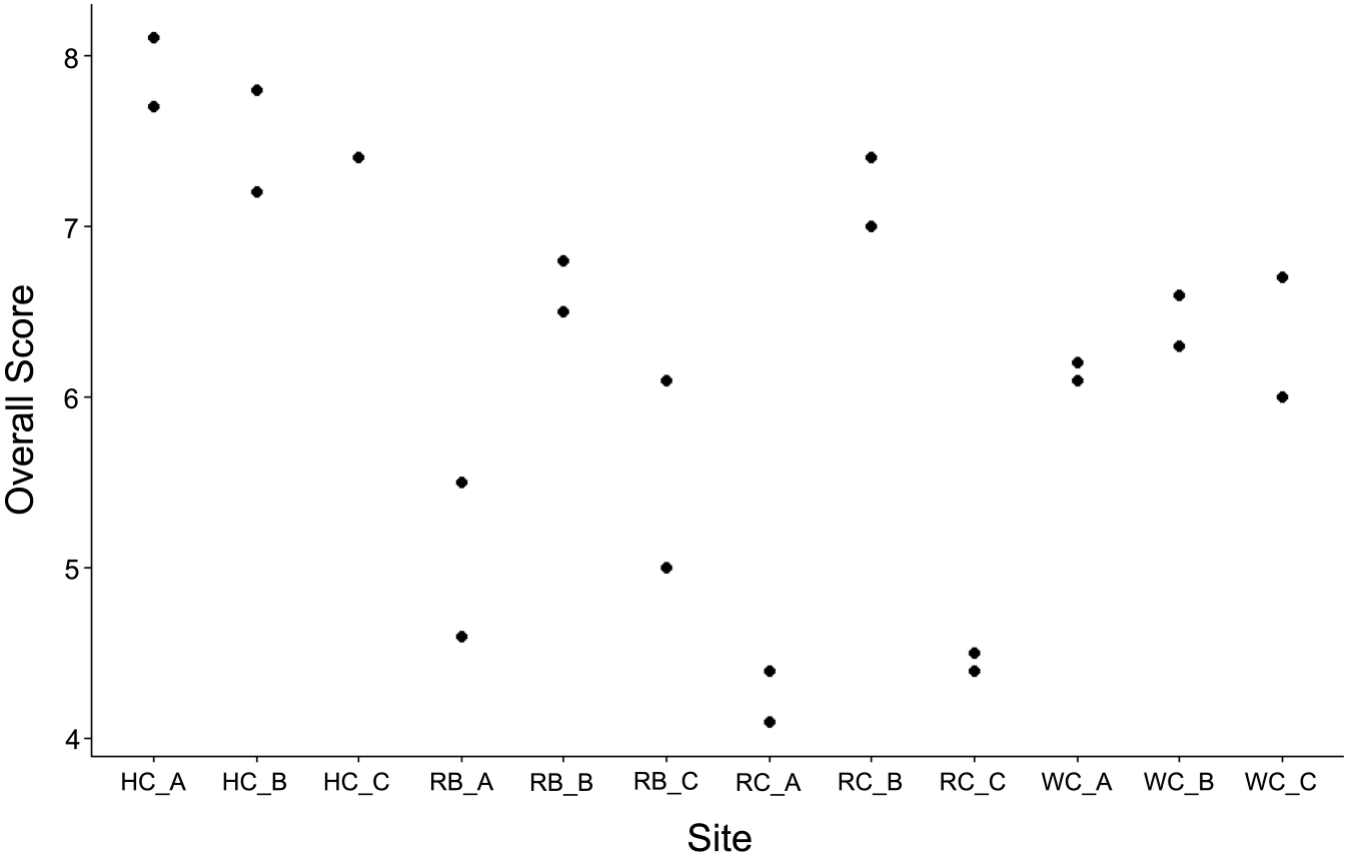
Overall SVAP score per site. Each stream site (4 streams: HC, House Creek; RB, Rocky Branch; RC, Richland Creek; WC, Walnut Creek; n = 3 sites per stream) was visited twice during 24 October through 14 November 2024. Overall scores for each site within a stream were calculated by averaging the scores for all 10 SVAP factors. Note that the overall score for HC site C was the same for both visits.

Overall SVAP scores per site were highest for House Creek, ranging from 7.2 to 8.1 among the three sites, indicating good stream habitat (Fig 3). Walnut Creek sites were also fairly consistent, with overall scores among sites ranging from 6.0 to 6.7. The other two streams were characterized by highly variable overall SVAP scores: Rocky Branch scores ranged from 4.6 to 6.8, with high variability in scores between the two sampling dates for the first and third sites. Low hydrologic alteration drove the higher overall score at RB_B compared to RB_A and RB_C. The high variability at RB_A and RB_C likely reflects variation in SVAP scoring. Only 0.25 cm of rain fell between October 24–31, so it is unlikely that differences in channel condition, hydrologic alteration, bank stability, and invertebrate cover were driven by storm runoff from Pullen Road and Morrill Drive, respectively.

Richland Creek overall scores ranged from 4.1 to 7.4 per site. While individual site scores were consistent across sample dates, sites were highly variable (≤ 4.5 for RC_A and RC_C, compared to 7–7.4 for RC_B). Site RC_A receives extensive surface runoff as well as inflow from a culvert from Wade Avenue during storm events, and the bank was reinforced in 2023 after a cave-in. These anthropogenic impacts drove low scores for riparian zone, hydrologic alteration, channel condition, bank stability, pools, and instream fish cover. Similarly, RC_C is immediately downstream of the northern egress of an extensive paved tunnel beneath Wade Avenue, which introduces substantial stormwater input and drives low scores for the same SVAP factors.

### Measured physical and water quality parameters

The four streams averaged 22–40 cm in depth and differed substantially in both flow rate (means 0.01 m/s in Rocky Branch to 0.23 m/s in Walnut Creek) and canopy cover (means 45% over Walnut Creek to 86% over House Creek) (Table 3). Mean temperatures among the streams varied from 13.6 to 19.1^o^C, with House Creek being coolest and Walnut Creek warmest.

**Table 3:** Measured physical and water quality parameters for the four urban streams (means ± standard error, ranges; data rounded to nearest significant decimal; satn. = saturation). Asterisks (*) denote values that exceed evaluation levels.

Variable	House Creek	Rocky Branch	Richland Creek	Walnut Creek
**Physical**				
Depth (cm)	22.00 ± 5.74 (7.7-45.0)	40.00 ± 2.60 (29-46)	29.00 ± 4.75 (17-46)	22.00 ± 4.05 (14-36)
Flow (m s^-1^)	0.15 ± 0.03 (0.8-0.3)	0.01 ± 0 (0-0.03)	0.11 ± 0.02 (0.064-0.22)	0.23 ± 0.06 (0.14-0.42)
Canopy cover (%)	85.54 ± 7.37 (62-100)	57.55 ± 6.87 (19-62)	69.36 ± 9.20 (49-99)	44.85 ± 6.61 (18-60)
Temperature (°C)	13.60 ± 0.92 (11.5-15.6)	17.83 ± 0.21 (17.2-18.5)	14.60 ± 1.40 (11.3-17.8)	19.10 ± 0.38 (18.1-20.0)
**Water Quality**				
DO (mg L^-1^)^a^	7.01 ± 0.20 (6.5-7.7)	6.67 ± 0.12 (6.2-7.1)	7.03 ± 0.38 (5.8-8.0)	5.86 ± 0.09* (5.5-6.1)
DO (% satn.)	67.40 ± 0.79 (65-71)	71.40 ± 1.98 (65-77)	70.00 ± 2.45 (61-77)	63.40 ± 0.95 (61-66)
Conductivity (µS/cm)	149.40 ± 1.75 (146-156)	180.13 ± 3.83 (163-190)	135.90 ± 2.10 (127-140)	75.10 ± 1.69 (69-78)
TDS (mg L^-1^)	106.00 ± 1.28 (103-111)	127.00 ± 2.59 (116-134)	96.90 ± 1.39 (91-99)	53.30 ± 1.10 (49 –56)
Turbidity (NTU)	2.48 ± 0.21 (1.9-3.3)	6.30 ± 0.83 (4.5-10.2)	6.91 ± 2.42 (3.2-18.6)	8.93 ± 0.97 (5.4-11.4)
pH	7.47 ± 0.07 (7.32-7.79)	7.21 ± 0.08 (7.00-7.56)	7.46 ± 0.08 (7.20-7.73)	6.84 ± 0.03 (6.74-6.94)
*Enterococcus*^b^ (CFU 100 mL^-1^)	91–479*	91–479*	1120*	1553*

^a^DO evaluation level ≥6.0 mg/L [32].

^b^*Enterococcus* evaluation level ≤ 35 CFU/100 mL [32].

Walnut Creek was substantially more acidic than the other streams, with a mean pH (6.84) ~0.4 to 0.6 units below the mean pH of the others. Stream turbidity was low when sampled in the absence of storm events. While DO concentrations, as measured during late morning, indicated adequate oxygen for beneficial fauna such as fish, the percent saturation suggests that biota in these streams sustain unhealthy conditions of low-oxygen stress during diel cycles [33]. *Enterococcus* densities ranged from 91 to 479 CFU 100 mL^-1^, except that samples collected on 7 November from Richland Creek and Walnut Creek measured 1120 and 1553 CFU 100 mL^-1^, respectively. The data suggest fecal contamination, as *Enterococcus* densities were well in excess of North Carolina water quality standard for freshwater human contact based on fecal coliform bacteria (200 CFU100 mL^-1^ as a geometric mean; single sample maximum of 400 CFU 100 mL^-1^) [32].

### Spearman’s Correlations (ρ): SVAP scores versus measured physical or water quality parameters

**Fig 4:**
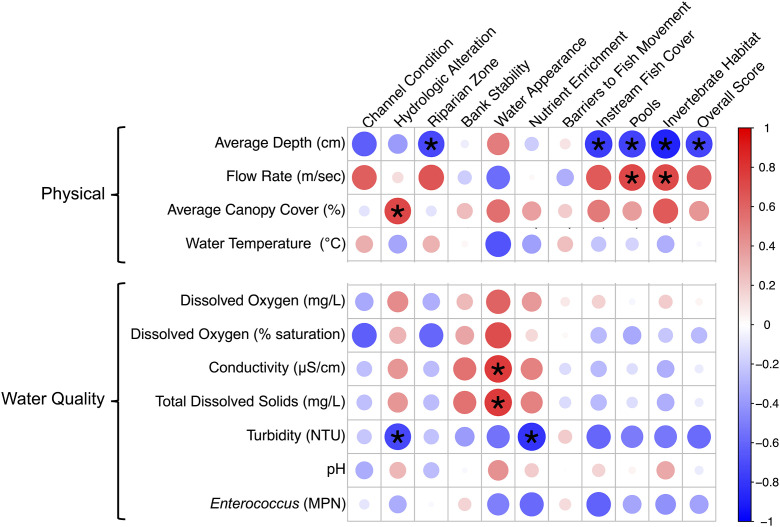
Spearman’s correlations (ρ) between visual metrics from SVAP metrics (horizontal axis) and measured physical (Phys.) characteristics or water quality (WQ) parameters (vertical axis), collected from the four urban streams. The size of each circle reflects the ρ value. Color indicates positive (red) or negative (blue) correlation; asterisks denote statistical significance (*p* < 0.05).

The measured physical environmental characteristics correlated strongly with several SVAP metrics (Fig 4, Table 4). Average depth correlated negatively with riparian zone (*ρ* = –0.715, *p* = 0.046), instream fish cover (*ρ* = –0.778, *p* = 0.023), pools (*ρ* = –0.747, *p* = 0.033), invertebrate habitat (*ρ* = –0.881, *p* = 0.004), and overall score (*ρ* = –0.738, *p* = 0.037). Flow rate correlated positively with pools (*ρ* = 0.723, *p* = 0.043) and invertebrate habitat (*ρ* = 0.714, *p* = 0.047). Average canopy cover correlated positively with hydrologic alteration (*ρ* = 0.723, *p* = 0.043).

**Table 4:** Significant Spearman correlations (ρ) between SVAP metrics and measured average physical or water quality parameters. Correlations in bold were predicted (Table 2). Asterisks (*) indicate that the direction of the correlation (positive or negative ρ value) supports the prediction.

SVAP metric	Measured physical characteristic	Water quality parameter	ρ	*p* value
Hydrologic alteration	Average canopy cover	-----	0.723	0.043
	-----	Turbidity	−0.723	0.043
Riparian Zone	Average depth	-----	−0.715	0.046
Water appearance	-----	**Conductivity**	0.771	0.025
	-----	**TDS**	0.771	0.025
Nutrient enrichment	-----	**Turbidity**	−0.805	0.016
Instream fish cover	**Average depth**	-----	−0.778	0.023
Pools	Average depth*	-----	−0.747	0.033
	Flow rate	-----	0.723	0.043
Invertebrate habitat	**Average depth**	-----	0.881	0.004
	Flow rate*	-----	0.714	0.047
Overall score	Average depth	-----	−0.738	0.037

In contrast, few strong correlations were found between measured water quality parameters and SVAP metrics. Turbidity was negatively correlated with hydrologic alteration (*ρ* = –0.723, *p* = 0.043) and nutrient enrichment (*ρ* = –0.805, *p* = 0.016). Conductivity (*ρ* = 0.771, *p* = 0.025) and TDS (*ρ* = 0.771, *p* = 0.025) were positively correlated with water appearance.

## Discussion

This study represents, to our knowledge, the first attempt to compare SVAP scores to measured physical and water quality data. The analyses yielded strong and significant SVAP correlations between both physical and water quality parameters (Table 4, Fig 4). However, among 121 potential correlations (11 SVAP scores x 11 water quality metrics), we detected only 12 significant relationships (9.9% of total potential utility). While our hypothesis was generally supported, physical characteristics are more robust predictors of SVAP metrics compared to water quality parameters (Table 4).

Average depth was the strongest predictor of SVAP scores, correlating negatively with riparian zone, instream fish cover, pools, and invertebrate habitat (Fig 4). This robust relationship likely reflects the ecological consequences of channelization and flashiness in urban streams. Interestingly, flow rate correlated positively with pools and invertebrate habitat, suggesting that water speed and depth incur distinct impacts on stream ecology. Canopy cover only correlated significantly with hydrologic alteration, similar to previous findings, confirming that it does not always correlate with greater stream health [34]. While TDS and conductivity correlated positively with water appearance, turbidity correlated negatively with hydrologic alteration and nutrient enrichment. Our results suggest that deeply incised channels and prevention of natural flooding events (that result in low hydrologic alteration) may increase the cloudiness of water but not its conductivity.

While riparian zones are crucial for filtering pollutants in runoff [35], we found no significant correlation between riparian zone and any physical or water quality scores except average depth – nor did *Enterococcus* measurements correlate with any SVAP scores (Fig 4). *Enterococcus* fecal bacteria are considered an important and reliable predictor of health risk associated with polluted recreational waters [36,37]. *Enterococcus* are used as fecal indicator bacteria because they originate from the guts of warm-blooded animals [38]; and increased levels of *Enterococcus* are associated with anthropogenic activity [39,40]. While we predicted that *Enterococcus* would correlate negatively with riparian zone, bank stability, water appearance, and positively with nutrient enrichment (Table 2), we detected no significant correlations between *Enterococcus* levels and any of the SVAP scores (Fig 4). Additional research is needed to increase sampling across time points, seasons, and additional streams to verify whether *Enterococcus* might correlate with SVAP scores.

Our findings underscore a fundamental need for water quality data as well as physical habitat data in assessments of stream health. Bjorkland et al. [2] found poor agreement between SVAP and NH_4_-N, NO_3_-N, and PO_4_-P for 9 sites assessed in Georgia. Carrie et al. [13] previously found that SVAP correlates strongly and positively with canopy cover across stream sites in Belize; but Principal Coordinate analysis relationships with depth, turbidity, pH, temperature, and DO were more ambiguous. Some major consulting firms currently use only habitat scores (the foundation of the SVAP) to assert that water quality is acceptable – despite the fact that physical appearance commonly does not translate to good water quality. For example, “water appearance” (color/murkiness) may not convey visible information about water quality except for relatively extreme physical conditions. Similarly, “nutrient enrichment” based on noticeable algal/plant growth does not distinguish whether the algal/plant growth is healthy or noxious and over-stimulated by nutrient pollution; benthic algae in particular can appear “overly abundant” when it is in fact healthy growth of beneficial taxa [41].

### Limitations

The few significant correlations between physical and water quality measures and SVAP scores in the current study could be attributed to several confounding variables, including limited sample size and variation due to weather events and SVAP observer subjectivity. Many pollutants enter streams shortly after a rainstorm begins, resulting in short-lived “spikes”. It often takes concerted, repeated sampling to catch these spikes, whereas storm-driven physical changes may be short-lived (e.g., water appearance) or persist longer (e.g., channel condition, bank stability) in the landscape. In addition, chemical stressors such as conductivity, bacterial contamination, and diel dissolved oxygen fluctuations are visibly imperceptible. We also noticed variation in overall SVAP scores between visits to each site (Fig 3), which may have resulted from weather events between visits or from inter-individual score variation. Researchers within randomized teams assigned different individual SVAP scores at each visit, and required extensive discussion to reach consensus scores for each site. As such, our limited sampling effort (2 dates at 4 streams in 1 season) may have prevented detection of genuine relationships.

Overall, we found that the SVAP alone is not a sufficient measure of stream health in urban streams, and is mostly useful as an introduction to water quality analysis or as an educational tool [2]. Because SVAP scoring involves subjective visual estimates, some variability among observers was expected. This underscores the importance of validation and calibration to ensure that the protocol can be applied reliably by students or citizen scientists.

### Future directions

The SVAP could be used together with one or more available water quality indices for stream assessment. There is a rich literature on the utility of various indices as composite indicators of river water quality that can be easily communicated and used as assessment, management, and predictive tools [42]. These water quality indices vary from inclusion of many to only a few parameters such as dissolved oxygen, conductivity, and turbidity [43].

Future studies with increased sample size across seasons could also measure score variability to assess observer bias and the reproducibility of SVAP scores across a diverse range of users. Additionally, we suggest an expansion and further integration of the glossary of the SVAP, as some technical terms were left undefined (e.g., “scarp”), adding complexity to vocabulary that potentially renders stream ecology less accessible to the public. We propose providing a definition at the first occurrence of each technical term in the SVAP, as well as visual training examples to help users score more accurately. While the SVAP2 includes clearer instructions with some pictures and tables [8], both protocols could be revised to include scores for each SVAP factor for the example pictures provided for each element, to ensure more consistent scoring between individuals in informal settings and across sites. In the meantime, water quality studies to inform the development or modification of regulations should continue to use traditional water quality measurements.

## Conclusions

**Fig 5:**
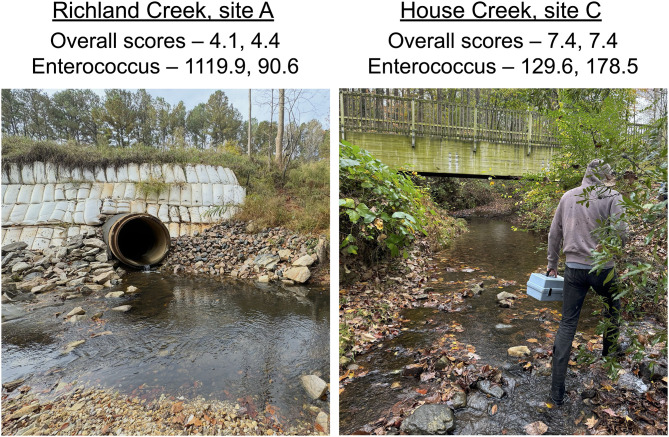
SVAP scores did not predict water quality, particularly *Enterococcus* (CFUs/100mL) levels. Here we feature Richland Creek site A (credit: **N.** Patterson) and House Creek site C (credit: **C.** McMurray), representative of relatively low and high SVAP scores, respectively (S1 Table).

The NRCS SVAP, based on physical features, is widely used as an initial screening technique to assess the general condition of wadeable streams, and mostly has been compared to macroinvertebrate indicators of stream health. General water quality has been inferred in the SVAP by the general physical attributes of color, turbidity, and obvious algal growth. We compared SVAP scores to actual water quality data and found that while correlations with measured physical characteristics were strong as expected (8 significant correlations, between 6 SVAP metrics and 3 physical parameters), only 3 water quality parameters correlated significantly with 3 SVAP scores. Notably, while *Enterococcus* and DO (mg/L) values exceeded evaluation standards, neither of these water quality parameters correlated with SVAP scores (Fig 5). Countering our initial expectation, the findings suggest that the SVAP should not be used to infer water quality conditions.

As suggested improvements to the SVAP, future studies with increased sample size could track score variability to improve assessment of observer bias and the reproducibility of SVAP scores across a diverse range of users (e.g., age groups, landowners, recreationists). The SVAP glossary could be amended to include definitions of some technical terms to strengthen general understanding about SVAP application. It would also be helpful to provide a definition at the first occurrence of each technical term in the SVAP, and visual training examples to help users score more accurately. SVAP2 includes clearer instructions with some pictures and tables [8], but protocols could be further revised to include scores for each SVAP factor for the example pictures of each element, to promote more consistent scoring between individuals in informal settings and across sites. These improvements would not change our central conclusion: The SVAP alone should not be used to infer water quality; inferences about water quality should be based on traditional water quality measurements.

## Supporting information

S1 Table Explanation of SVAP guidelines [7] and examples of assigned scores, as interpreted in the current study. (DOCX)
