# Supplementary material for: Evaluation of stream visual assessment protocol with measured water quality parameters in urban streams
Source: PLoS One. 2026 Jun 25;21(6):e0351972. doi: 10.1371/journal.pone.0351972 (PMC13298787; doi:10.1371/journal.pone.0351972)
Supplement: S1 Table — (DOCX) [file pone.0351972.s001.docx]

Supplementary Table 1. Explanation of SVAP guidelines (7) and examples of assigned scores, as interpreted in the current study.

| **SVAP metric** | **SVAP guideline** | **Example low score** | **Example high score** |
| --- | --- | --- | --- |
| Channel condition | “Often, development in the area results in changes to this meandering pattern and the flow of a stream. These changes in turn may affect the way a stream naturally does its work, such as the transport of sediment and the development and maintenance of habitat for fish, aquatic insects, and aquatic plants.” | RC_A (scored 2,2)  Site is 3-5 meters from dirt trail but far bank is directly below Wade Avenue / I-440W, and had to be reinforced with sandbagging in 2023 | RB_B (scored 10,8)  Stream site located 10-20 meters from dirt trail; bank intact and slopes gently to sand beaches |
| Hydrologic alteration | “Bankfull flows, as well as flooding, are important to maintaining channel shape and function (e.g. sediment transport) and maintaining the physical habitat for animals and plants.” | RC_C (scored 2, 3)  Steep banks and deep channel incision – erosion evident, but no evidence of flooding | RC_B (scored 9,8)  Gentle bank slopes and sandy beaches accommodate water fluctuations and flooding |
| Riparian zone | “[T]he width of the natural vegetation zone from the edge of the active channel out onto the floodplain.” | RB_A (scored 2, 2)  Stream site located within 2 meters of paved trail; sparse grass and visible bare dirt | WC_C (scored 10,10)  No trail; 10-15 meters of dense vegetation standing 1-1.5 meters high with trees throughout |
| Bank stability | “[T]he existence of or the potential for detachment of soil from the upper and lower stream banks and its movement into the stream.” | RC_C (scored 3, 2)  Steep banks with bare tree roots exposed by extensive erosion | HC_C (scored 7,7)  Gradual slopes, rounded banks, secured by grasses and ferns growing all the way to the water, with trees growing ~1 meter from stream |
| Water appearance | “[Comparison of] turbidity, color, and other visual characteristics with a healthy or reference stream.” | WC_A (scored 5, 5)  Stream bed was visible in depths less than 1 meter, but could not be seen in deeper pools | HC_A (scored 10, 8)  Water was clear and colorless, even in deeper pools |
| Nutrient enrichment | “Nutrient enrichment is often reflected by the types and amounts of aquatic vegetation in the water. High levels of nutrients (especially phosphorus and nitrogen) promote an over-abundance of algae and floating and rooted macrophytes.” | RB_B (scored 3, 5)  Large amount of algae seen growing on rocks, and water had a slight green tint from suspended algae, though no aquatic vegetation was present | RB_A (scored 9, 8)  Very little algae seen growing on the surface of rocks (thin layer) but not suspended in the water |
| Barriers to fish movement | “If sufficiently high, these barriers may prevent the movement or migration of fish, deny access to important breeding and foraging habitats, and isolate populations of fish and other aquatic animals.” | WC_C (scored 7, 6)  Several large rocks and a partially submerged pipe ~0.5 meter in diameter, perpendicular to the stream flow | RB_A (scored 10,10)  Nothing in the water blocking flow or fish movement |
| Instream fish cover | “The potential for the maintenance of a healthy fish community and its ability to recover from disturbance is dependent on the variety and abundance of suitable habitat and cover available.” | RC_A (scored 3, 3)  Identified 3 items providing cover including undercut bank and two large rocks | HC_B (scored 10, 10)  Counted >7 items providing fish cover including pools, tree cover, varying channel depth, rocks, fallen trees |
| Pools | “Pools are important resting and feeding sites for fish. A healthy stream has a mix of shallow and deep pools.” | RB_C (scored 1, 1)  No pools; stream shallow and streambed entirely visible | HC_B (scored 7, 7)  Several bends in the stream with deeper pools |
| Invertebrate habitat | “Stable substrate is important for insect/invertebrate colonization. Substrate refers to the stream bottom, woody debris, or other surfaces on which invertebrates can live.” | RB_A (scored 1, 5)  On first visit, no habitat was observed. However, between visits a storm event introduced woody debris into the stream, establishing substrate identified on second visit. | HC_C (scored 10, 10)  Counted >5 instances of large rocks and woody debris from fallen trees |
